# Assessment of myocardial deformation by CMR tissue tracking reveals left ventricular subclinical myocardial dysfunction in patients with gynecologic cancer undergoing chemotherapy

**DOI:** 10.3389/fonc.2025.1464368

**Published:** 2025-02-03

**Authors:** Kai Tao, Lu Ye, Yan-Jia-Ni Xu, Meng-Xi Yang, Ru-Tie Yin, Qing-Li Li, Xiao-Juan Lin, Ke-Min Li, Liang Song, Yu Ma, Lan Zhong, Ying Hu, Hua-Yan Xu, Dan-Qing Wang, Ying-Kun Guo

**Affiliations:** ^1^ Department of Radiology, Key Laboratory of Birth Defects and Related Diseases of Women and Children of Ministry of Education, West China Second University Hospital, Sichuan University, Chengdu, China; ^2^ West China School of Medicine, West China Hospital, Sichuan University, Chengdu, China; ^3^ Department of Ultrasound, Key Laboratory of Birth Defects and Related Diseases of Women and Children of Ministry of Education, West China Second University Hospital, Sichuan University, Chengdu, China; ^4^ Department of Cardiology, Laboratory of Cardiac Structure and Function at Institute of Cardiovascular Diseases, and Cardiac Structure and Function Research Key Laboratory of Sichuan Province, West China Hospital, Sichuan University, Chengdu, China; ^5^ Department of Radiology, Sichuan Cancer Hospital & Institute, Sichuan Cancer Center, School of Medicine, University of Electronic Science and Technology of China, Chengdu, China; ^6^ Department of Gynaecology and Obstetrics, Key Laboratory of Birth Defects and Related Diseases of Women and Children of Ministry of Education, West China Second University Hospital, Sichuan University, Chengdu, China

**Keywords:** cardiac magnetic resonance, cardiotoxicity, tissue tracking, left ventricular deformation, neoplasm

## Abstract

**Background:**

Chemotherapy-induced cardiotoxicity is a concern for patients with gynecologic cancer. This study aimed to assess left ventricular (LV) myocardial deformation in patients with gynecologic cancer undergoing chemotherapy and to investigate the association between myocardial deformation and chemotherapy factors.

**Methods:**

Cardiac magnetic resonance (CMR) was performed to assess LV deformation parameters using CMR tissue tracking based on cine images. Serum myocardial injury biomarker were measured. Deformation parameters were compared between healthy controls and patients. Changes in deformation were assessed as chemotherapy progressed. Correlations between LV deformation parameters, clinical characteristics, and serum myocardial injury biomarkers were also analyzed.

**Results:**

A total of 86 patients with gynecologic cancer and 30 normal controls were included. Among the patients, 41 completed CMR follow-up with a median interval of 6 months. Compared to the controls, patients exhibited lower absolute value of global radial strain (GRS) (37.30 ± 8.94% vs. 44.32 ± 8.44%), global circumferential strain (GCS) (-22.12 ± 3.05% vs. -24.08 ± 2.13%) and global longitudinal strain (GLS) (median -15.72% [IQR-17.13 to -13.58%] vs. -17.51 ± 2.00 %) (all *p* < 0.05). Patients with preserved LV ejection fraction (LVEF) also showed impaired global strain (all *p* < 0.05). GRS (39.71 ± 8.09% vs. median 30.56% [IQR 26.52 to 38.15%]; *p* = 0.001), GCS (-23.45 ± 2.09% vs. median -19.71% [IQR -21.71 to -19.10%]; *p* < 0.001) and GLS (-16.17 ± 2.42% vs. median -12.12% [IQR -14.10 to -8.53%]; *p*< 0.001) further decreased as the number of chemotherapy cycles increased during follow-up (all *p* < 0.05). Multivariate analysis showed that GCS was independently associated with the number of chemotherapy regimens (Standard regression coefficient [β] = 0.397, *p* < 0.001).

**Conclusions:**

Myocardial deformation is more sensitive than LVEF in detecting subclinical left ventricular dysfunction in patients with gynecologic cancer undergoing chemotherapy. GCS was associated with the number of chemotherapy regimens.

## Introduction

As the survival rates and lifespans of cancer patients continue to increase, chemotherapy-related cardiotoxicity has become a critical concern (1, 2). Gynecologic cancers pose a significant threat to women’s health, and their incidence is rising (3). Chemotherapy is a key treatment of gynecologic cancers (4), but several commonly used chemotherapy drugs have been reported to cause cardiotoxicity and myocardial dysfunction (5). Therefore, early assessment and monitoring of cardiac function are essential for patients with gynecologic cancer undergoing chemotherapy.

Clinically, left ventricular ejection fraction (LVEF), which reflects left ventricular systolic function, remains the gold standard for assessing ventricular function during and after cancer therapy. However, LVEF may be insufficient for detecting subclinical cardiac dysfunction (6, 7).

Cardiac magnetic resonance (CMR) myocardial strain values, which provide a non-invasive quantitative measurement for analyzing heart deformation, have emerged as a more promising and sensitive index for estimating ventricular systolic function (8, 9). CMR tissue tracking is a promising contrast-free quantitative method which is based on CMR cine sequence images to quantify global and segmental myocardial strain (9). It has been shown to offer diagnostic and prognostic value beyond LVEF in patients with coronary artery disease, dilated cardiomyopathy, heart failure and valvular diseases (10–13). Moreover, previous studies have applied CMR tissue tracking to monitor chemotherapy-associated cardiotoxicity (14–18), demonstrating that CMR global longitudinal strain (GLS) and global circumferential strain (GCS) can detect and predict early cardiac dysfunction. However, there is a lack of research on myocardial deformation in patients with gynecologic cancer undergoing chemotherapy. Therefore, this study aimed to assess left ventricular (LV) myocardial deformation in patients with gynecologic cancer undergoing chemotherapy using CMR tissue tracking and to investigate the association between LV myocardial deformation and chemotherapy factors.

## Methods

### Study design

This study is part of a registered clinical research (registration No. ChiCTR-DDD-17013450, http://www.chictr.org.cn). It was approved by the institutional research ethics board of the authors’ hospital, and written informed consent was obtained from each participation prior to the investigation. In this single-center prospective cohort study, we screened patients with gynecologic cancer undergoing chemotherapy in the Division of Chemotherapy and Radiotherapy in the Department of Gynecology from September 2018 to August 2022. They all satisfied with the including criteria: (1) diagnosed (initially diagnosed or recurrent) with gynecologic cancer; (2) undergoing chemotherapy; and (3)between the ages of 18 and 75 years. The exclusion criteria included (1) preexisting cardiovascular diseases, including coronary heart disease, cardiomyopathy, valvular heart disease, congenital heart disease and pericardial disease; (2) history of cardiotoxic medication or chest radiation for other diseases; (3) CMR contraindications; and (4) poor CMR cine images quality. CMR was performed in intermission of chemotherapy or progression free interval. This study also recruited age-matched female healthy volunteers as healthy control subjects. Healthy controls with preexisting cardiovascular risk factors or disease, contraindications to CMR or poor quality of CMR cine images were excluded. Patients with LVEF ≥ 55% were classified as preserved LVEF (PLVEF) group, and patients with LVEF < 55% were classified as reduced LVEF (RLVEF) group.

### Cardiac MRI

Patients and normal controls were examined in the supine position using a 3.0 T scanner (MAGNETOM Skyra, Siemens Healthineers, Erlangen, Germany) equipped with an 18-channel receiver coil. All images were obtained during breath-holding in end-expiration, and electrocardiographic gating was employed. To quantify the cardiac structure and function, 8 to 12 continuous sections were obtained from the mitral valve level to LV apex in the short-axis view using a balanced steady-state free precession pulse sequence: echo time (TE) = 1.22 ms, temporal resolution (TR) = 39.34 ms, flip angle = 40°, slice thickness = 8 mm, matrix = 208 × 208 pixels, and field of view (FOV) = 340 × 284 mm^2^. The vertical two-chamber long axis and horizontal four-chamber cine series were scanned using the same sequences used with the short-axis images.

### Imaging analysis

**Figure 1:**
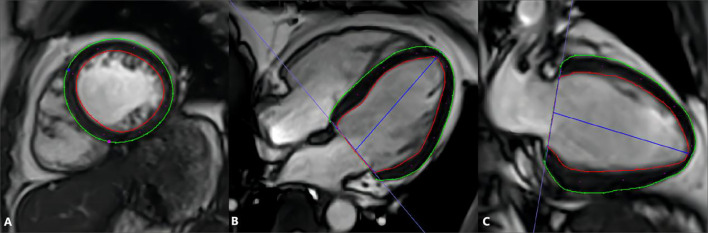
Measurement of MR tissue tracking by Cvi42. Tracing the endo- (red curve) and epicardial (green curve) borders on the short axis **(A)**, 4-chamber **(B)**, and 2-chamber long axis **(C)** cine images; define the short axis reference point (blue and red points) at the insertion of the right ventricle and left ventricle on the short axis slice.

Cine MRI data were analyzed by professional post-processing software (Cvi42, version 5.11; Circle Cardiovascular Imaging Inc, Calgary, Canada) to detect LVEF and LV deformation (**Figure 1**). According to the common division for LV myocardial strain with 16 segmentations (19), the apical cap was not included in the range of strain analysis. A set of short-axis, four-chamber and long-axis two-chamber slices were loaded into the tissue tracking module. All endocardial and epicardial borders were traced manually. The deformation parameters were then automatically determined by the software. Tissue tracking deformation parameters included LV radial, circumferential, longitudinal strain and strain rate (SR). Strain parameters included global strain and regional strain at basal, middle and apical levels. In addition, the corresponding polar maps and curve graphs of different strain were obtained.

### Examination of serum myocardial injury biomarkers

Serum myocardial injury biomarkers which include cardiac troponin I (cTnI), myohemoglobin (Myo), creatine kinase (CK) and cardiac isoenzyme of creatine kinase (CK-MB) were measured on the day of CMR examination. The serum samples were measured by Siemens ADVIA Centaur XPT to gain the value of cTnI, Myo, CK and CK-MB.

### Statistical analysis

All statistics were analyzed by IBM SPSS 25.0 (Armonk, NY) and GraphPad Prism 7.0 (San Diego, CA). For continuous variables, the Shapiro-Wilk test was first used to check for normality. If the data followed a normal distribution, they were presented as mean with standard deviation; if not, they were presented as median with interquartile range (IQR). Categorical variables were expressed as counts and percentages of the total. For the comparison of continuous variables between the patient group and the normal control group, if the data followed a normal distribution and had homogeneity of variance, an independent t-test was used; otherwise, the rank sum test was employed for comparison. For the comparison of continuous variables among PLVEF group, RLVEF group and normal controls, if the data followed a normal distribution and had homogeneity of variance, ordinary one-way ANOVA analysis was employed; otherwise, the Kruskal–Wallis test was employed. In the patients who completed CMR follow-up, the parameters between two scans were compared. For the comparison of CMR continuous variable data from the same patient at two scans, if the data followed a normal distribution and have homogeneity of variance, a paired t-test was used; otherwise, the Wilcoxon signed-rank test was applied. For the correlation analysis of two continuous variables, if both variables follow a normal distribution, Pearson’s correlation analysis is used; otherwise, Spearman’s correlation analysis is applied. Subsequently, the variables were entered into a multivariate linear regression model to identify the factors independently associated with the deformation parameters. *P* values < 0.05 were considered statistically significant.

## Results

### General characteristics

The general characteristics of patients with gynecologic cancer and healthy control subjects are showed in **Table 1**. A total of 86 patients with gynecologic cancer who underwent chemotherapy and 30 healthy control subjects were included. The mean age of patients with gynecologic cancers was 50.5 years (IQR 45.0-56.0 years). Among the 86 patients, 48 (55.8%) were diagnosed with ovarian cancer, 14 (16.3%) were diagnosed with fallopian tube cancer, 20 (23.3%) with uterine neoplasm and 4 (4.6%) with trophoblastic tumor. Among the patients, the cardiovascular risk factors were hypertension in 6 patients (7.0%) and diabetes in 3 patients (3.5%); 13 patients (15.2%) had a history of cardioprotective medication use. The median number of chemotherapy cycles was 6.0 (IQR 3.0-11.0). The median number of chemotherapy regimens was 1.0 (1.0, 2.0). 50 patients (58.1%) received only one regimen, among which the taxol plus platinum regimen was the most commonly used (n = 42, 48.8%); 21 patients (24.4%) received two regimens; and 15 patients (17.4%) received three or more regimens.

**Table 1:** General characteristics of healthy control subjects and patients with gynecologic cancer.

	Healthy Control Subjects (n=30)	Patients with Gynecologic Cancer (n = 86)	*p* value
Demographics			
Age at CMR (years)	42.5 (36.0, 57.0)	50.5 (45.0, 56.0)	0.072
Heart rate (beats/min)	70.6 (66.2, 74.0)	77.0 (70.0, 90.1)	0.002*
Body surface area (m^2^)	1.51 ± 0.13	1.56 ± 0.14	0.077
Body mass index (kg/m^2^)	22.46 ± 3.13	23.50 ± 3.61	0.164
Systolic blood pressure (mm Hg)	119.6 ± 9.2	118.0 ± 11.4	0.492
Diastolic blood pressure (mm Hg)	73.5 (66.3, 85.0)	79.0 (70.0, 85.0)	0.120
CVD risk factors (n, %)			
Hypertension	—	6 (7.0%)	—
Diabetes	—	3 (3.5%)	—
Smoke	—	1 (1.2%)	—
Cardioprotective Medication (n, %)			
Calcium antagonist	—	5 (5.8%)	—
ARB	—	2 (2.3%)	—
ACEI	—	1 (1.2%)	—
Diuretics	—	1 (1.2%)	—
Beta-blockers	—	4 (4.6%)	—
Cancer diagnosis (n, %)			
Ovarian cancer	—	48 (55.8%)	—
Fallopian tube cancer	—	14 (16.3%)	—
Uterine neoplasm	—	20 (23.3%)	—
Trophoblastic tumor	—	4 (4.6%)	—
Cancer onset (n, %)			
Initial diagnosed	—	50 (58.1%)	—
Recurrence	—	36 (41.9%)	—
**Operation (n, %)**	—	82 (95.3%)	—
Chemotherapy factors			
Number of drug types	—	2.5 (2.0, 4.0)	—
Number of chemotherapy cycles	—	6.0 (3.0, 11.0)	—
chemotherapy duration (month)†	—	10.5 (3.0, 26.3)	
Number of chemotherapy regimens	—	1.0 (1.0, 2.0)	—
One regimen	—	50 (58.1%)	—
Taxol + Platinum	—	42 (48.8%)	—
Anthracycline + Platinuml	—	4 (4.7%)	—
Bleomycin + Platinuml	—	2 (2.3%)	—
Others		2 (2.3%)	
Two regimens	—	21 (24.4%)	—
Three or more regimens	—	15 (17.4%)	—

Data were presented as median (IQR), mean ± SD or number (percentage).

*P <0.05.

†the time elapsed from the initial chemotherapy to the cardiac magnetic resonance.

CVD, cardiovascular disease; ARB, angiotensin receptor blocker; ACEI, angiotensin converting enzyme inhibitor.

Of the 86 patients enrolled, 41 (47.67%) completed the CMR follow-up. During a median interval of 6 months (IQR 3–9 months) between two scans, these patients had undergone a median of 3 cycles (IQR 2–5 cycles) of chemotherapy.

### Deformation parameters in healthy controls subjects and patients with gynecologic cancer

**Figure 2:**
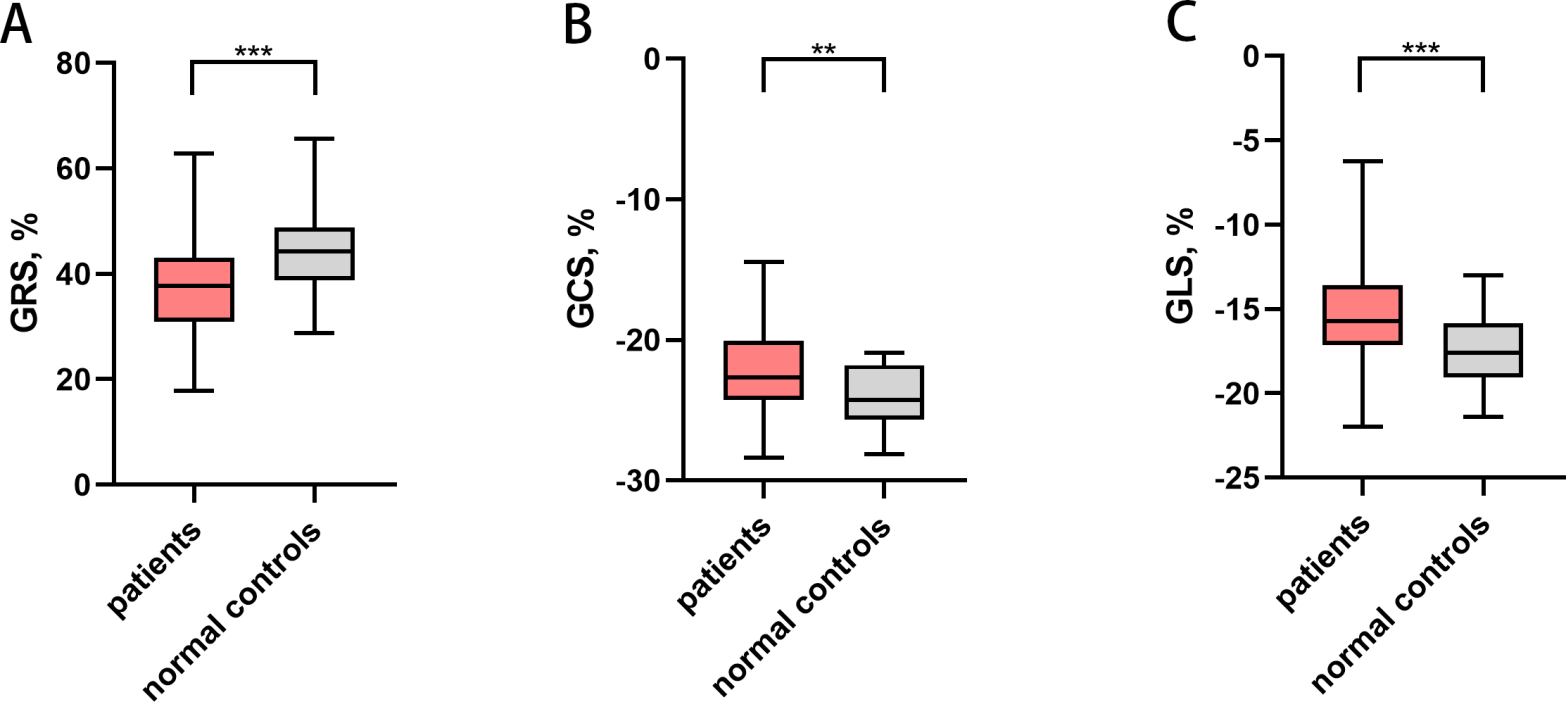
Left ventricular global strain in patients with gynecologic cancer and normal controls. ** P<0.01, *** P<0.001. **(A)** Left ventricular GRS, **(B)** Left ventricular GCS, **(C)** Left ventricular GLS. Patients had lower absolute value of GRS, GCS and GLS. GRS, global radial strain; GCS, global circumferential strain; GLS, global longitudinal strain.

No significant difference in LVEF between the healthy control subjects and patients with gynecologic cancer (*p* = 0.594) was observed. The absolute value of LV global radial strain (GRS) (37.30 ± 8.94% vs. 44.32 ± 8.44%; *p* < 0.001), GCS (-22.12 ± 3.05% vs. -24.08 ± 2.13%; *p* = 0.002) and GLS (median -15.72% [IQR-17.13 to -13.58%] vs. -17.51 ± 2.00 %; *p* < 0.001) were all lower in patients compared with controls (**Figure 2**). In addition, most LV regional strain parameters demonstrated significant differences between patients and controls (**Table 2**). The absolute value of global radial diastolic strain rate (RDSR) (-2.48 1/s [IQR -3.01 to -2.01 1/s] vs. median -3.25 1/s [IQR -3.73 to -3.00 1/s; *p* < 0.001) and global circumferential diastolic strain rate (CDSR) (1.26 ± 0.26 1/s vs. 1.50 ± 0.31 1/s; *p* < 0.001) was lower in patients compared with controls (**Table 2**). Patients received only taxol plus platinum regimen (n = 42) also had lower absolute value of GRS (37.40 ± 7.61% vs. 44.32 ± 8.44%; *p* = 0.001), GCS (-22.50 ± 2.83% vs. -24.08 ± 2.13%; *p* = 0.012), GLS (median -15.87% [IQR-17.07 to -13.92%] vs. median -17.60% [IQR -19.05 to -15.84%]; *p* = 0.001), RDSR(-2.40 1/s [IQR -3.03 to -1.92 1/s] vs. median -3.25 1/s [IQR -3.73 to -3.00 1/s; *p* < 0.001) and CDSR(1.26 ± 0.28 1/s vs. 1.50 ± 0.31 1/s; *p* = 0.002) compared with controls. There were no significant differences of LV global strain and strain rate between anthracycline-treated patients and nonanthracycline-treated patients.

**Table 2:** CMR deformation parameters of healthy control subjects and patients with gynecologic cancer.

	Healthy control subjects (n=30)	Patients (n=86)	P value
**LVEF (%)**	63.91 ± 5.25	63.22 ± 6.27	0.594
Strain (%)			
BRS	53.60 ± 10.88	45.41(39.48, 50.67)	0.001*
BCS	-21.00 ± 2.07	-19.94 ± 2.87	0.066
BLS	-15.74 (-17.30, -14.47)	-14.58 (-16.84, -11.41)	0.081
MRS	43.15 ± 8.25	36.55 ± 9.80	0.001*
MCS	-24.39 ± 2.19	-22.43 ± 3.19	0.004*
MLS	-16.72 ± 2.73	-15.40 (-16.91, -13.08)	0.008*
ARS	43.08 ± 15.82	35.44 (25.56, 46.26)	0.044*
ACS	-27.69 (-30.11, -24.89)	-25.86 (-27.65, -22.80)	0.004*
ALS	-20.16 ± 1.98	-18.17 (-19.47, -16.38)	<0.001*
GRS	44.32 ± 8.44	37.30 ± 8.94	<0.001*
GCS	-24.08 ± 2.13	-22.12 ± 3.05	0.002*
GLS	-17.51 ± 2.00	-15.72 (-17.13, -13.58)	<0.001*
Strain rate (1/s)			
RSSR	2.42 (2.01, 2.79)	2.24 (1.79, 2.68)	0.152
CSSR	-1.19 ± 0.18	-1.17 (-1.33, -1.04)	0.912
LSSR	-0.90 (-1.07, -0.77)	-0.92 (-1.17, -0.74)	0.474
RDSR	-3.25 (-3.73, -3.00)	-2.48 (-3.01, -2.01)	<0.001*
CDSR	1.50 ± 0.31	1.26 ± 0.26	<0.001*
LDSR	1.06 ± 0.23	0.94 (0.80, 1.20)	0.192

Data were presented as median (IQR) or mean ± SD.

*P <0.05.

BRS, basal radial strain; BCS, basal circumferential strain; BLS, basal longitudinal strain; MRS, mid radial strain; MCS, mid circumferential strain; MLS, mid longitudinal strain; ARS, apical radial strain; ACS, apical circumferential strain; ALS, apical longitudinal strain; GRS, global radial strain; GCS, global circumferential strain; GLS, global longitudinal strain; RSSR, global radial systolic strain rate; CSSR, global circumferential systolic strain rate; LSSR, global longitudinal systolic strain rate; RDSR, global radial diastolic strain rate; CDSR, global circumferential diastolic strain rate; LDSR, global longitudinal diastolic strain rate.

**Figure 3:**
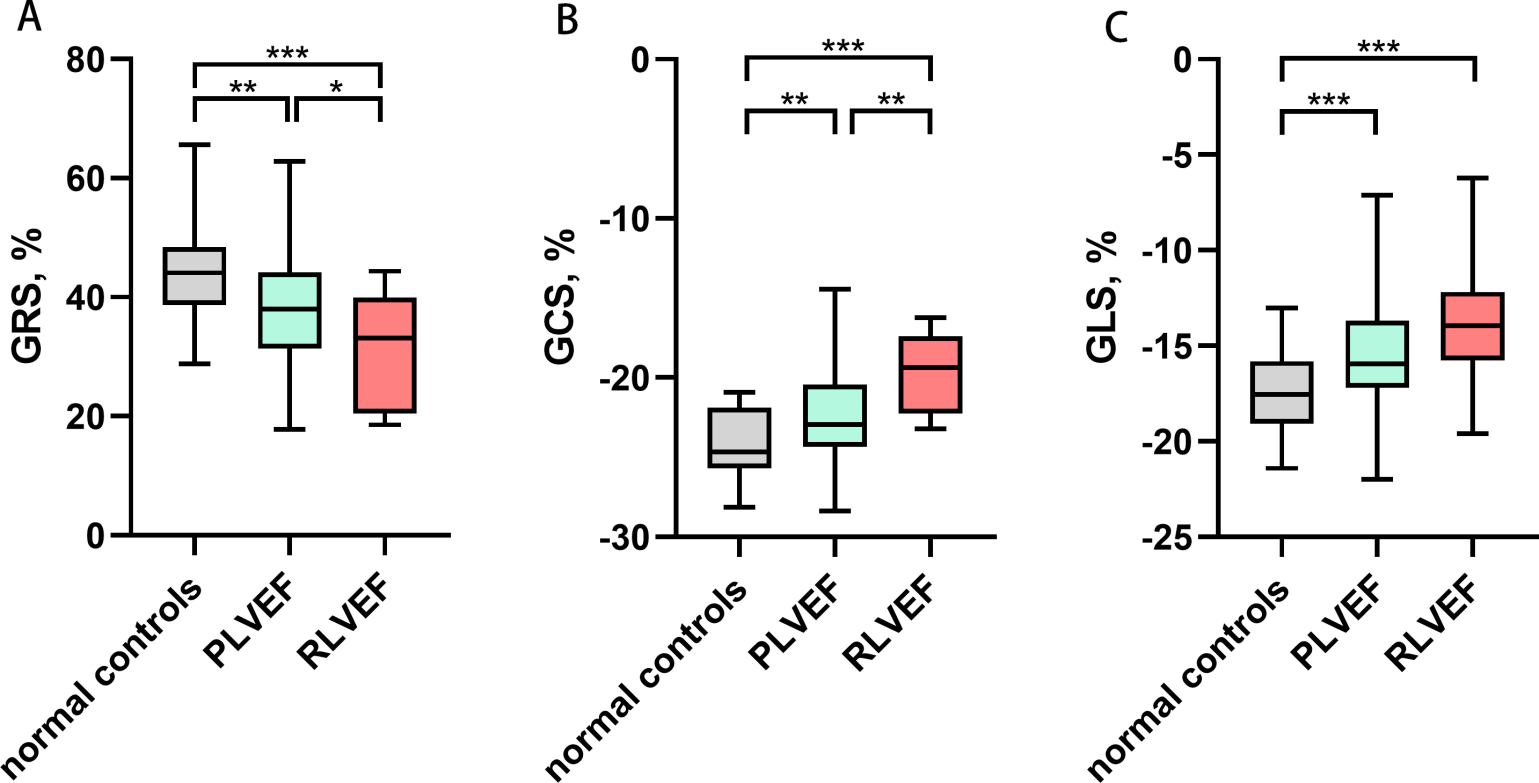
Comparisons of left ventricular global strain among normal controls, PLVEF group and RLVEF group. * P<0.05, ** P<0.01, *** P<0.001. **(A)** Left ventricular GRS, **(B)** Left ventricular GCS, **(C)** Left ventricular GLS. The absolute value of GRS, GCS and GLS decreased in both PLVEF group and RLVEF group compared with normal controls. GRS, global radial strain; GCS, global circumferential strain; GLS, global longitudinal strain; LVEF, left ventricular ejection fraction; PLVEF, preserved left ventricular ejection fraction; RLVEF, reduced left ventricular ejection fraction.

Among 86 patients with gynecologic cancer, 77 patients were classified as PLVEF group, and 9 patients were classified as RLVEF group. We found a decrease in the absolute value of GRS (37.99 ± 8.65% vs. 44.32 ± 8.44%; *p* = 0.007), GCS (-22.42 ± 2.98% vs. -24.08 ± 2.13 %; *p* = 0.004), GLS (median -15.94% [IQR -17.19 to -13.68%] vs. -17.51 ± 2.00%; *p* = 0.001) and several regional strain parameters in PLVEF group compared with control group (**Figure 3**). Moreover, the absolute value of RDSR(median -2.49 1/s [IQR -3.03 to -2.07 1/s] vs. median -3.25 1/s [IQR -3.73 to-3.00 1/s; *p*< 0.001) and CDSR (1.29 ± 0.25 1/s vs. 1.51 ± 0.29 1/s; *p* < 0.001) were lower in PLVEF group compared with control group (**Table 3**).

**Table 3:** CMR deformation parameters of healthy control subjects, PLVEF group and RLVEF group.

	Healthy control subjects (n=30)	PLVEF group (n=77)	RLVEF group (n=9)	P value
**LVEF (%)**	63.91 ± 5.25	64.56 ± 4.95	51.79 ± 4.56	
Strain (%)				
BRS	53.60 ± 10.88	46.40 (39.58, 51.71)*	41.15 (38.69, 44.74)*	0.003
BCS	-21.00 ± 2.07	-20.24 ± 2.78	-17.36 ± 2.32*#	0.002
BLS	-15.74 (-17.30, -14.47)	-14.82 (-16.64,-11.39)	-13.29 (-18.67,-10.82)	0.245
MRS	43.15 ± 8.25	37.20 ± 9.71*	31.16 ± 9.29*	0.002
MCS	-24.39 ± 2.19	-22.65 ± 3.18*	-20.49 ± 2.79*#	0.001
MLS	-16.72 ± 2.73	-15.41 (-16.93,-13.35)*	-13.94 (-17.14, -9.76)*	0.031
ARS	43.08 ± 15.82	36.74 ± 15.72	30.17 ± 18.24	0.066
ACS	-27.69 (-30.11, -24.89)	-26.10 (-27.92,-23.36)*	-22.89 (-26.82,-18.34)*	0.001
ALS	-20.16 ± 1.98	-18.45 (-19.98,-16.61)*	-16.48 (-18.22,-9.75)*	<0.001
GRS	44.32 ± 8.44	37.99 ± 8.65*	31.55 ± 9.73*#	<0.001
GCS	-24.08 ± 2.13	-22.42 ± 2.98*	-19.54 ± 2.54*#	<0.001
GLS	-17.51 ± 2.00	-15.94 (-17.19,-13.68)*	-13.94 (-15.76,-12.19)*	0.001
Strain rate (1/s)				
RSSR	2.42 (2.01, 2.79)	2.25 (1.88, 2.70)	1.62 (1.46, 2.30)	0.073
CSSR	-1.19 ± 0.18	-1.17 (-1.37, -1.08)	-0.95 (-1.28, -0.89)	0.077
LSSR	-0.90 (-1.07, -0.77)	-0.91 (-1.18, -0.74)	-0.97 (-1.08, -0.81)	0.814
RDSR	-3.25 (-3.73, -3.00)	-2.49 (-3.03,-2.07)*	-1.65 (-2.56, -1.07)*	<0.001
CDSR	1.51 ± 0.29	1.29 ± 0.25*	1.00 ± 0.19*#	<0.001
LDSR	1.06 ± 0.23	0.94 (0.80, 1.21)	0.92 (0.79, 1.12)	0.444

Data were presented as median (IQR) or mean ± SD.

* P <0.05 versus healthy control subjects; # *p*<0.05 versus PLVEF group.

RLVEF, reduced left ventricular ejection fraction, PLVEF, preserved left ventricular ejection fraction; BRS, basal radial strain; BCS, basal circumferential strain; BLS, basal longitudinal strain; MRS, mid radial strain; MCS, mid circumferential strain; MLS, mid longitudinal strain; ARS, apical radial strain; ACS, apical circumferential strain; ALS, apical longitudinal strain; GRS, global radial strain; GCS, global circumferential strain; GLS, global longitudinal strain; RSSR, global radial systolic strain rate; CSSR, global circumferential systolic strain rate; LSSR, global longitudinal systolic strain rate; RDSR, global radial diastolic strain rate; CDSR, global circumferential diastolic strain rate; LDSR, global longitudinal diastolic strain rate.

### Changes in deformation parameters during chemotherapy

**Figure 4:**
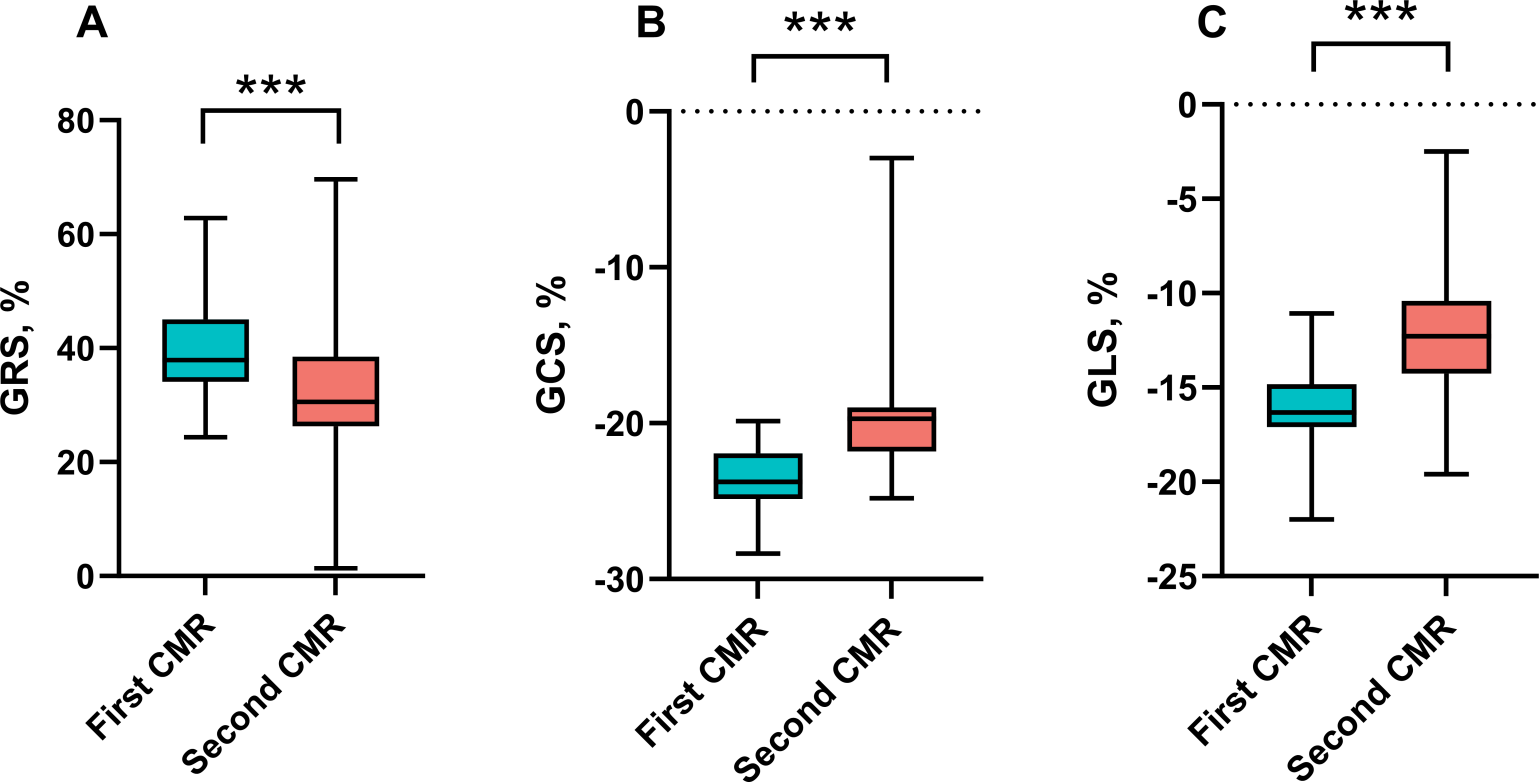
Left ventricular global strain in the patients between two CMR scans. *** P<0.001 . **(A)** Left ventricular GRS, **(B)** Left ventricular GCS, **(C)** Left ventricular GLS. The absolute value of GRS, GCS and GLS decreased significantly at the second CMR scan during follow-up. GRS, global radial strain; GCS, global circumferential strain; GLS, global longitudinal strain.

In the 41 patients who completed follow-up, the absolute value of GRS (39.71 ± 8.09% vs. median 30.56% [IQR 26.52 to 38.15%]; *p* = 0.001), GCS (-23.45 ± 2.09% vs. median -19.71% [IQR -21.71 to -19.10%]; *p* < 0.001) and GLS (-16.17 ± 2.42% vs. median -12.12% [IQR -14.10 to -8.53%]; *p*< 0.001) were all decreased at follow-up (**Figure 4**). The decrease in the absolute value of several regional strains was also observed (**Table 4**). Moreover, the absolute value of RDSR (median -2.33 1/s [IQR -3.00 to -1.98 1/s] vs. median -1.89 1/s [IQR -2.48 to -1.43 1/s]; *p* = 0.006) and CDSR (1.32 ± 0.25 1/s vs. 1.13 ± 0.27 1/s; *p* = 0.002) decreased at follow-up.

**Table 4:** Myocardial deformation parameters between two CMR scans.

	First CMR scan (n = 41)	Second CMR scan (n = 41)	P Value
BRS	45.25 (38.86, 50.67)	49.62 ± 18.27	0.236
BCS	-21.25 ± 2.32	-19.03 (-20.27, -17.31)	<0.001*
BLS	-14.47 ± 3.42	-13.28 (-16.08, -11.28)	0.223
MRS	39.49 ± 9.98	31.26 (24.48, 37.09)	0.002*
MCS	-23.58 ± 2.71	-19.79 (-22.38, -18.65)	<0.001*
MLS	-15.49 ± 3.06	-13.94 (-15.56, -12.33)	0.026*
ARS	39.86 ± 13.91	23.02 ± 15.15	<0.001*
ACS	-26.67 ± 2.63	-21.73 (-24.68, -19.27)	<0.001*
ALS	-18.62 ± 2.19	-9.03 (-16.18, -4.85)	<0.001*
GRS	39.71 ± 8.09	30.56 (26.52, 38.15)	0.001*
GCS	-23.45 ± 2.09	-19.71 (-21.71, -19.10)	<0.001*
GLS	-16.17 ± 2.42	-12.12 (-14.10, -8.53)	<0.001*
RSSR	2.34 (1.86, 2.70)	1.95 (1.61, 2.50)	0.478
CSSR	-1.26 ± 0.19	-1.09 (-1.29, -0.98)	0.022 *
LSSR	-0.89 (-1.10, -0.75)	-0.97 (-1.20, -0.72)	0.988
RDSR	-2.33 (-3.00, -1.98)	-1.89 (-2.48, -1.43)	0.006 *
CDSR	1.32 ± 0.25	1.13 ± 0.27	0.002 *
LDSR	0.91 (0.81, 1.13)	0.97 (0.70, 1.14)	0.252

Data were presented as median (IQR) or mean ± SD.

*P <0.05.

BRS, basal radial strain; BCS, basal circumferential strain; BLS, basal longitudinal strain; MRS, mid radial strain; MCS, mid circumferential strain; MLS, mid longitudinal strain; ARS, apical radial strain; ACS, apical circumferential strain; ALS, apical longitudinal strain; GRS, global radial strain; GCS, global circumferential strain; GLS, global longitudinal strain; RSSR, global radial systolic strain rate; CSSR, global circumferential systolic strain rate; LSSR, global longitudinal systolic strain rate; RDSR, global radial diastolic strain rate; CDSR, global circumferential diastolic strain rate; LDSR, global longitudinal diastolic strain rate.

### Association between LV deformation parameters and general characteristics

The correlation analysis between deformation parameters and demographics showed that GLS was positively correlated with systolic blood pressure (r = 0.213, *p* = 0.049). We also did the correlation analysis between deformation parameters and chemotherapy-related factors, including the number of chemotherapy cycles and the number of chemotherapy regimens. Univariate analysis showed that GCS was positively correlated with the number of chemotherapy regimens (r = 0.290, *p* = 0.007). In the multivariate analysis which was adjusted for demographical confounders, GCS was independently associated with the number of chemotherapy regimens (Standard regression coefficient [β] = 0.397, *p* < 0.001) (**Table 5**).

**Table 5:** Univariable and multivariable association analysis of global strain and general characteristics.

	GRS				GCS				GLS			
	Univariable r	P value	Multivariable β	P value	Univariable r	P value	Multivariable β	P value	Univariable r	P value	Multivariable β	P value
Systolic blood pressure	-0.181	0.100	-0.192	0.101	0.054	0.623	0.099	0.369	0.213*	0.049	0.157	0.186
age	0.129	0.243	0.082	0.470	-0.197	0.070	-0.101	0.352	-0.013	0.909	0.046	0.690
BMI	-0.165	0.135	-0.165	0.165	0.103	0.345	0.040	0.724	0.086	0.431	0.091	0.453
heart rate	-0.075	0.506	-0.003	0.979	0.019	0.866	-0.076	0.493	0.053	0.638	-0.004	0.976
The number of chemotherapy regimens	-0.141	0.201	-0.096	0.400	0.290*	0.007	0.397*	<0.001	0.062	0.570	0.068	0.560

*P <0.05.

GRS, global radial strain; GCS, global circumferential strain; GLS, global longitudinal strain; BMI, body mass index.

### Correlation between LV deformation parameters and LVEF and serum myocardial injury biomarkers

Correlation analysis showed that all global strain parameters and most strain rate parameters were correlated with LVEF (*p* < 0.05) (**Table 6**).

**Table 6:** Correlation between LV deformation parameters and LVEF and serum myocardial injury biomarkers.

	LVEF		CK-MB		Myo	
	r	P Value	r	P Value	r	P Value
GRS	0.596	<0.001*	-0.047	0.698	-0.121	0.303
GCS	-0.627	<0.001*	0.167	0.154	0.298	0.009*
GLS	-0.383	0.001*	0.241	0.038*	0.213	0.064
RSSR	0.553	<0.001*	0.022	0.856	-0.105	0.369
CSSR	-0.457	<0.001*	0.137	0.245	0.342	0.003*
LSSR	-0.283	0.008*	0.297	0.010*	0.243	0.034*
RDSR	-0.549	<0.001*	-0.024	0.839	0.200	0.083
CDSR	0.482	<0.001*	0.075	0.526	-0.258	0.025*
LDSR	0.211	0.051	0.070	0.551	-0.079	0.496

*P<0.05. LVEF, left ventricular ejection fraction; CK-MB, cardiac isoenzyme of creatine kinase; Myo, myohemoglobin; GRS, global radial strain; GCS, global circumferential strain; GLS, global longitudinal strain; RSSR, global radial systolic strain rate; CSSR, global circumferential systolic strain rate; LSSR, global longitudinal systolic strain rate; RDSR, global radial diastolic strain rate; CDSR, global circumferential diastolic strain rate; LDSR, global longitudinal diastolic strain rate.

The results of serum myocardial injury biomarkers showed that all patients had normal cTnI (0~0.06 ug/L) and Myo (0~110 ug/L); 85 patients had normal CK-MB (0~5 ug/L) and CK (39~192 U/L). Among the serum biomarkers, CK-MB was correlated with GLS (r = 0.241, *p* = 0.038) and global longitudinal systolic strain rate (LSSR) (r = 0.297, *p* = 0.010); and Myo was correlated with GCS (r = 0.298, *p* = 0.009), global circumferential systolic strain rate (CSSR) (r = 0.342, *p* = 0.003), LSSR (r = 0.243, *p* = 0.034) and CDSR (r = -0.258, *p* = 0.025) (**Table 6**).

## Discussion

The main findings of this study were as follows: (1) Patients with gynecologic cancer undergoing chemotherapy showed significant decrease in LV deformation parameters compared with control subjects. Moreover, LV deformation parameters further decreased as the chemotherapy cycles increased during follow-up. The above results indicated that chemotherapy could result in LV deformation impairment in patients with gynecologic cancers. (2) The deformation parameters were correlated with LVEF. In patients with preserved LVEF, the deformation parameters were impaired, which indicated that LV dysfunction had already occurred even when the LVEF was within the normal range. (3) GCS was independently associated with the number of chemotherapy regimens, indicating that the circumferential strain impairment was obvious with the increase of chemotherapy regimens. (4) Some LV deformation parameters were correlated with serum myocardial injury biomarkers.

Antineoplastic therapy is frequently complicated by the development of cardiotoxicity (5). Detection of myocardial dysfunction in patients with cancers is essential to predict prognosis and improve the survival rates and quality of life in patients. CMR tissue tracking have shown promise for quantitatively assessing subclinical cardiac dysfunction (20). Our study found that the absolute value of global strain was lower in cancer patients undergoing chemotherapy compared with healthy controls. It was consistent with the result of a prior study reported by Lunning MA et al. who investigated 10 adult patients treated with anthracycline-based chemotherapy by CMR and showed that GCS (*p* = 0.046) and GLS (*p* = 0.035) of the patients were lower than that of controls (21). Our study also found that deformation parameters further decreased as the chemotherapy cycles increased during follow-up, which was similar with the results of several prior studies. Drafts BC et al. showed that the mid-wall circumferential strain changed (-17.7 ± 0.4% vs. -15.1 ± 0.4%, *p* = 0.0003) within 6 months after low to moderate doses of anthracycline-based chemotherapy (22). Jolly MP et al. found that the mean mid-wall circumferential strain fell from −18.8 ± 2.9% to −17.6 ± 3.1%(*p* = 0.001)after 3 months of chemotherapy (18). Jordan JH et al. showed that mid-wall Eulerian circumferential strain declined from -17.99% to -17.23% (*p* = 0.0052) (17). Similarly, our study showed the decrease of the absolute value of MCS. Lunning MA et al. found that GCS was significantly decreased in patients after 3 months of chemotherapy (*p* = 0.018) (21); and Jordan JH et al. found that GLS decreased from -15.44% to -14.79% (*p* = 0.0069) (17). Our study also showed the decrease of the absolute value of GCS and GLS.

In previous studies, the cancer types of the subjects were mainly breast cancer and lymphoma (14, 15, 21). Different from previous studies, the subjects of our study were patients with gynecologic cancer. To the best of our knowledge, the present research is the first prospective study to focus on LV myocardial deformation parameters associated with chemotherapy for gynecologic cancer. In our previous cross-sectional study, we focused on chemotherapy effect on myocardial fibrosis markers in patients with gynecologic cancers and low cardiovascular risk and found that these patients had impaired LV GLS compared with healthy controls. However, in this prospective study, all global strain were impaired. This is likely because this study did not exclude individuals with cardiovascular risk factors.

CMR myocardial tissue tracking using balanced steady-state free precession (bSSFP) cine imaging has been developed to meet the need for fast and quantitative assessment of myocardial strain analysis. It serves as the CMR-equivalent of speckle-tracking echocardiography while addressing some of the limitations of echocardiography, such as acoustic window constraints and low spatial resolution (23, 24). Validated against myocardial tagging, CMR tissue tracking can conveniently be performed using bSSFP imaging as part of a routine CMR scan, requiring no additional sequences (25, 26). Furthermore, CMR tissue tracking contribute to detect early changes in myocardial mechanics in pathology (subclinical) with normal or preserved ejection fraction (27). In our study, the PLVEF group demonstrated impaired LV deformation parameters, even when LVEF remained within the normal range. This suggests that LV deformation parameters can detect LV dysfunction earlier than LVEF. Additionally, a significant correlation was observed between LV deformation parameters and LVEF, reinforcing the notion that LV deformation parameters are more sensitive than LVEF in detecting subclinical LV dysfunction.

We studied the association between LV deformation parameters and chemotherapy-related factors. Our previous study has found that the number of chemotherapy cycles was positively related to extracellular volume fraction and negatively related to intracellular mass indexed (28). Unlike our previous study, this study revealed the association between the number of chemotherapy regimens and GCS. Our finding indicated that the LV circumferential strain impairment was obvious with the increase of chemotherapy regimens.

The chemotherapeutic drugs can damage myocyte’s sarcolemma which caused release of bioactive markers into extracellular environment (29). However, conventional biomarkers cannot reflect chemotherapy-induced myocardial toxicity early (29). One previous study collected blood samples of lung cancer patients who received chemotherapy and found that chemotherapy didn’t cause obvious elevation of CK-MB (30). Similarly, our study showed that almost all patients had normal serum biomarkers. However, the impairment of deformation parameters of patients was found in our study. We also found that some LV deformation parameters were correlated with serum biomarkers. These findings indicated that LV deformation parameter impairment might be associated with myocardial injury and that deformation parameters could reflect myocardial injury earlier than serum biomarkers.

Anthracyclines can contribute to dose-related cardiotoxicity and left ventricular dysfunction (31–33). In our study, we compared the deformation parameters in anthracycline-treated patients and nonanthracycline-treated patients and found that there was no significant difference. We also found that patients received only taxol plus platinum regimen had impaired global deformation parameters compared with control subjects. These findings indicated that nonanthracycline chemotherapy could also cause subclinical LV dysfunction.

Our study had several limitations. Firstly, this study was conducted at a single center with a relatively small sample size, a short follow-up period, and a low follow-up rate.The limited follow-up period restricts the ability to observe long-term outcomes like the development of heart failure. This could lead to an underestimation of the true incidence and introduce bias by under-representing patients with late-onset outcomes. To mitigate this limitation, future studies with longer follow-up periods or supplemental observational data are necessary for a more comprehensive assessment. Secondly, not all inpatients were invited to participate, and a selection bias cannot be ignored. Thirdly, there were several chemotherapy regimens used in this population, so the respective effects of these drugs require further elucidation. However, we believe this reflects the real-world situation of the gynecologic malignancy population.

## Conclusions

In conclusion, patients receiving chemotherapy for gynecological cancer develop subclinical LV dysfunction during treatment. Myocardial deformation is a more sensitive measure than LVEF for detecting subclinical dysfunction in patients with gynecologic cancer undergoing chemotherapy. Additionally, global circumferential strain was associated with the number of chemotherapy regimens in these patients.

## Data Availability

The raw data supporting the conclusions of this article will be made available by the authors, without undue reservation.

## References

[1] Alvarez-Cardona JA Ray J Carver J Zaha V Cheng R Yang E. Cardio-oncology education and training: JACC council perspectives. J Am Coll Cardiol. (2020) 76:2267–81. doi: 10.1016/j.jacc.2020.08.079 PMC8174559 33153587

[2] Lenneman CG Sawyer DB. Cardio-oncology: an update on cardiotoxicity of cancer-related treatment. Circ Res. (2016) 118:1008–20. doi: 10.1161/CIRCRESAHA.115.303633 26987914

[3] Bhatla N Denny L. FIGO cancer report 2018. Int J Gynaecol Obstet. (2018) 143 Suppl 2:2–3. doi: 10.1002/ijgo.12608 30306587

[4] Armstrong DK Alvarez RD Bakkum-Gamez JN Barroilhet L Behbakht K Berchuck A. Ovarian cancer, version 2.2020, NCCN clinical practice guidelines in oncology. J Natl Compr Canc Netw. (2021) 19:191–226. doi: 10.6004/jnccn.2021.0007 33545690

[5] Yeh ET Bickford CL. Cardiovascular complications of cancer therapy: incidence, pathogenesis, diagnosis, and management. J Am Coll Cardiol. (2009) 53:2231–47. doi: 10.1016/j.jacc.2009.02.050 19520246

[6] Makavos G Ikonomidis I Palios J Rigopoulos A Katogiannis K Parissis J. Cardiac imaging in cardiotoxicity: a focus on clinical practice. Heart Fail Rev. (2021) 26:1175–87. doi: 10.1007/s10741-020-09952-w 32306221

[7] Cannizzaro MT Inserra MC Passaniti G Celona A D’angelo T Romeo P. Role of advanced cardiovascular imaging in chemotherapy-induced cardiotoxicity. Heliyon. (2023) 9:e15226. doi: 10.1016/j.heliyon.2023.e15226 37095987 PMC10121465

[8] Pedrizzetti G Claus P Kilner PJ Nagel E. Principles of cardiovascular magnetic resonance feature tracking and echocardiographic speckle tracking for informed clinical use. J Cardiovasc Magn Reson. (2016) 18:51. doi: 10.1186/s12968-016-0269-7 27561421 PMC5000424

[9] Xu J Yang W Zhao S Lu M. State-of-the-art myocardial strain by CMR feature tracking: clinical applications and future perspectives. Eur Radiol. (2022) 32:5424–35. doi: 10.1007/s00330-022-08629-2 35201410

[10] Eitel I Stiermaier T Lange T Rommel KP Koschalka A Kowallick JT. Cardiac magnetic resonance myocardial feature tracking for optimized prediction of cardiovascular events following myocardial infarction. JACC Cardiovasc Imaging. (2018) 11:1433–44. doi: 10.1016/j.jcmg.2017.11.034 29454776

[11] Romano S Judd RM Kim RJ Kim HW Klem I Heitner JF. Feature-tracking global longitudinal strain predicts death in a multicenter population of patients with ischemic and nonischemic dilated cardiomyopathy incremental to ejection fraction and late gadolinium enhancement. JACC Cardiovasc Imaging. (2018) 11:1419–29. doi: 10.1016/j.jcmg.2017.10.024 PMC6043421 29361479

[12] Karamitsos TD Francis JM Myerson S Selvanayagam JB Neubauer S. The role of cardiovascular magnetic resonance imaging in heart failure. J Am Coll Cardiol. (2009) 54:1407–24. doi: 10.1016/j.jacc.2009.04.094 19796734

[13] Zito C Carerj S Todaro MC Cusma-Piccione M Caprino A Di Bella G. Myocardial deformation and rotational profiles in mitral valve prolapse. Am J Cardiol. (2013) 112:984–90. doi: 10.1016/j.amjcard.2013.05.031 23800550

[14] Calvillo-Argüelles O Thampinathan B Somerset E Shalmon T Amir E Steve Fan CP. Diagnostic and prognostic value of myocardial work indices for identification of cancer therapy-related cardiotoxicity. JACC Cardiovasc Imaging. (2022) 15:1361–76. doi: 10.1016/j.jcmg.2022.02.027 35926895

[15] Houbois CP Nolan M Somerset E Shalmon T Esmaeilzadeh M Lamacie MM. Serial cardiovascular magnetic resonance strain measurements to identify cardiotoxicity in breast cancer: comparison with echocardiography. JACC Cardiovasc Imaging. (2021) 14:962–74. doi: 10.1016/j.jcmg.2020.09.039 33248962

[16] Ong G Brezden-Masley C Dhir V Deva DP Chan KKW Chow CM. Myocardial strain imaging by cardiac magnetic resonance for detection of subclinical myocardial dysfunction in breast cancer patients receiving trastuzumab and chemotherapy. Int J Cardiol. (2018) 261:228–33. doi: 10.1016/j.ijcard.2018.03.041 29555336

[17] Jordan JH Sukpraphrute B Meléndez GC Jolly MP D’agostino RB Jr. Hundley WG. Early myocardial strain changes during potentially cardiotoxic chemotherapy may occur as a result of reductions in left ventricular end-diastolic volume: the need to interpret left ventricular strain with volumes. Circulation. (2017) 135:2575–7. doi: 10.1161/CIRCULATIONAHA.117.027930 PMC5508602 28630272

[18] Jolly MP Jordan JH Meléndez GC Mcneal GR D’agostino RB Jr. Hundley WG. Automated assessments of circumferential strain from cine CMR correlate with LVEF declines in cancer patients early after receipt of cardio-toxic chemotherapy. J Cardiovasc Magn Reson. (2017) 19:59. doi: 10.1186/s12968-017-0373-3 28768517 PMC5541737

[19] Lang RM Bierig M Devereux RB Flachskampf FA Foster E Pellikka PA. Recommendations for chamber quantification: a report from the American Society of Echocardiography’s Guidelines and Standards Committee and the Chamber Quantification Writing Group, developed in conjunction with the European Association of Echocardiography, a branch of the European Society of Cardiology. J Am Soc Echocardiogr. (2005) 18:1440–63. doi: 10.1016/j.echo.2005.10.005 16376782

[20] Smiseth OA Torp H Opdahl A Haugaa KH Urheim S. Myocardial strain imaging: how useful is it in clinical decision making? Eur Heart J. (2016) 37:1196–207. doi: 10.1093/eurheartj/ehv529 PMC4830908 26508168

[21] Lunning MA Kutty S Rome ET Li L Padiyath A Loberiza F. Cardiac magnetic resonance imaging for the assessment of the myocardium after doxorubicin-based chemotherapy. Am J Clin Oncol. (2015) 38:377–81. doi: 10.1097/COC.0b013e31829e19be 24192805

[22] Drafts BC Twomley KM D’agostino R Jr. Lawrence J Avis N Ellis LR. Low to moderate dose anthracycline-based chemotherapy is associated with early noninvasive imaging evidence of subclinical cardiovascular disease. JACC Cardiovasc Imaging. (2013) 6:877–85. doi: 10.1016/j.jcmg.2012.11.017 PMC3745801 23643285

[23] Hor KN Baumann R Pedrizzetti G Tonti G Gottliebson WM Taylor M. Magnetic resonance derived myocardial strain assessment using feature tracking. J Vis Exp. (2011) (48): 2356. doi: 10.3791/2356 21372778 PMC3074463

[24] Hundley WG Bluemke DA Finn JP Flamm SD Fogel MA Friedrich MG. ACCF/ACR/AHA/NASCI/SCMR 2010 expert consensus document on cardiovascular magnetic resonance: a report of the American College of Cardiology Foundation Task Force on Expert Consensus Documents. Circulation. (2010) 121:2462–508. doi: 10.1161/CIR.0b013e3181d44a8f PMC3034132 20479157

[25] Hor KN Gottliebson WM Carson C Wash E Cnota J Fleck R. Comparison of magnetic resonance feature tracking for strain calculation with harmonic phase imaging analysis. JACC Cardiovasc Imaging. (2010) 3:144–51. doi: 10.1016/j.jcmg.2009.11.006 20159640

[26] Wu L Germans T Güçlü A Heymans MW Allaart CP Van Rossum AC. Feature tracking compared with tissue tagging measurements of segmental strain by cardiovascular magnetic resonance. J Cardiovasc Magn Reson. (2014) 16:10. doi: 10.1186/1532-429X-16-10 24450803 PMC3926943

[27] Claus P Omar AMS Pedrizzetti G Sengupta PP Nagel E. Tissue tracking technology for assessing cardiac mechanics: principles, normal values, and clinical applications. JACC Cardiovasc Imaging. (2015) 8:1444–60. doi: 10.1016/j.jcmg.2015.11.001 26699113

[28] Ye L Wang DQ Yang MX Li QL Luo H Lin XJ. Chemotherapy effect on myocardial fibrosis markers in patients with gynecologic cancer and low cardiovascular risk. Front Oncol. (2023) 13:1173838. doi: 10.3389/fonc.2023.1173838 37614506 PMC10442931

[29] Urbanová D Urban L Carter A Maasova D Mladosievicova B. Cardiac troponins–biochemical markers of cardiac toxicity after cytostatic therapy. Neoplasma. (2006) 53:183–90. 16652186

[30] Demkow U Biatas-Chromiec B Stelmaszczyk-Emmel A Radzikowska E Wiatr E Radwan-Rohrenschef P. The cardiac markers and oxidative stress parameters in advanced non-small cell lung cancer patients receiving cisplatin-based chemotherapy. Ejifcc. (2011) 22:6–15. 27683384 PMC4975326

[31] Bovelli D Plataniotis G Roila F. Cardiotoxicity of chemotherapeutic agents and radiotherapy-related heart disease: ESMO Clinical Practice Guidelines. Ann Oncol. (2010) 21 Suppl 5:v277–82. doi: 10.1093/annonc/mdq200 20555097

[32] Henriksen PA. Anthracycline cardiotoxicity: an update on mechanisms, monitoring and prevention. Heart. (2018) 104:971–7. doi: 10.1136/heartjnl-2017-312103 29217634

[33] Senkus E Kyriakides S Ohno S Penault-Llorca F Poortmans P Rutgers E. Primary breast cancer: ESMO Clinical Practice Guidelines for diagnosis, treatment and follow-up. Ann Oncol. (2015) 26 (Suppl 5):v8–30. doi: 10.1093/annonc/mdv298 26314782

