# Supplementary material for: Assessment of myocardial deformation by CMR tissue tracking reveals left ventricular subclinical myocardial dysfunction in patients with gynecologic cancer undergoing chemotherapy
Source: Front Oncol. 2025 Feb 3;15:1464368. doi: 10.3389/fonc.2025.1464368 (PMC11830601; doi:10.3389/fonc.2025.1464368)
Supplement: Supplementary file 1 [file Table1.docx]

**Table S1. Review of myocardial deformation assessed by CMR in cancer patients after chemotherapy or before chemotherapy exposure**

| **First author** | **year** | **n** | **Age (years)** | **women, %** | **cancer** | **treatment** | **results** |
| --- | --- | --- | --- | --- | --- | --- | --- |
| Drafts BC et al. [1] | 2013 | 53 patients | 50 ± 2 | 58 | breast cancer leukemia lymphoma | anthracycline | left ventricular strain (−17.7±0.4 to −15.1±0.4; p=0.0003) increased within 6 months after low to moderate doses of anthracycline. |
| Nakano S et al. [2] | 2016 | 9 patients | 62.3 ± 12.6 | 100 | breast cancer | trastuzumab | Global left ventricular peak systolic longitudinal and circumferential strains were significantly decreased at 6 months (LS, -21.1 ± 1.7% [baseline] vs. -19.5 ± 1.0% [6 months], p = 0.039, and CS, -23.4 ± 1.8% [baseline] vs. -21.6 ± 2.5% [6 months], p = 0.036). |
| Jolly MP et al. [3] | 2017 | 72 patients | 53.8 ± 14.2 | 67 | breast cancer [39%], lymphoma [49%], or sarcoma [12%]) | Anthracycline, Antimicrotubule agents, Alkylating agents, Tyrasine-Kinase inhibitors, Antimetabolites | The results from these automated measures averaged −18.8 ± 2.9 at baseline and −17.6 ± 3.1 at 3 months (p = 0.001).The correlation between strain from cine imaging and LVEF was r = −0.61 (p < 0.0001). In addition, the 3-month changes in LV strain and LVEF were correlated (r = −0.49; p < 0.0001). The correlation between cine and tagged derived assessments of strain was r = 0.23; p = 0.01. |
| Ong G et al. [4] | 2018 | 41 patients | 52 ± 11 | 100 | breast cancer | trastuzumab | Compared to baseline, GLS and GCS decreased significantly at 6 months (p = 0.024 and < 0.001, respectively) and 12 months (p = 0.002 and < 0.001, respectively) with an increase in LV end-diastolic volume, but not at 18 months. |
| Gong IY et al. [5] | 2019 | 41 patients | 52 (11) | 100 | breast cancer | trastuzumab | There was a significant decline in peak systolic strain GLS and GCS at 6 months (p = 0.024 and p < 0.001, respectively) and 12 months (p = 0.002 and p < 0.001, respectively), followed by recovery at 18 months.Early diastolic strain rates did not significantly change over 18 months (p > 0.10), while global radial diastolic strain rate was marginally significant at 12 months (p = 0.021). |
| Lambert J et al. [6] | 2020 | 30 patients and 30 controls | 53.8 ± 7.1 (No CTRCD) 53.0 ± 8.0 (CTRCD) | 100 | breast cancer | anthracyclines and trastuzumab | Patients with cancer therapeutics-related cardiac dysfunction demonstrated larger mean temporal changes in all parameters compared with those without: 2D-GLS: 1.9% versus 0.7%, 2D-GCS: 2.5% versus 2.2%; CMR-GCS: 2.7% versus 1.6%; and CMR-GLS: 2.1% versus 1.4%, with overlap in 95% CI for 2D-LVEF, 2D-GCS, CMR-GLS and CMR-GCS. |
| Houbois CP et al. [7] | 2020 | 125 patients | 50.9 ± 9.0 | 100 | HER2+ early stage breast cancer | anthracycline/trastuzumab | Twenty-eight percent of patients developed CTRCD by CMR and 22% by 2DE. A 15% relative reduction in 2DE-GLS increased the CTRCD odds by 133% at subsequent follow-up, compared with 47%/50% by tagged-CMR GLS/ GCS and 87% by FT-GCS. CMR and 2DE-LVEF and indexed left ventricular end-systolic volume were also associated with subsequent CTRCD |
| Barbosa MF et al. [8] | 2021 | 18 patients and 12 controls | 57.6 ± 14.6 (anthracycline) 48.1 ± 12.2 (control) | 86 (anthracycline) 92 (control) | non-Hodgkin lymphoma | anthracycline | Compared with controls, anthracycline group showed impaired LV global early diastolic circumferential strain rate [53.5%/s ± 19.3 vs 72.2%/s ± 26.7, p = 0.022], early diastolic longitudinal strain rate [40.4%/s ± 13.0 vs 55.9%/s ± 17.8, p = 0.006] and early diastolic radial strain rate [- 114.4%/s ± 37.1 vs - 170.5%/s ± 48.0, p < 0.001]. |
| Bouwer NI et al. [9] | 2021 | 47 patients | 57.0 (50.0, 63.0) | 100 | breast cancer | trastuzumab | During trastuzumab treatment, GLS increased with 0.24%-points per month  (95% CI 0.14%-points, 0.32%-points; p < 0.001) and  GRS declined with − 0.68%-points per month (95% CI  − 0.94%-points, 0.42%-points; p < 0.001). |
| Labib D et al. [10] | 2021 | 381 patients and 102 controls | 53.8 ± 13.4 | 79 | active breast cancer lymphoma | before cardiotoxic chemotherapy exposure (anthracyclines and/or trastuzumab) | The only observed exception was GLS amplitude in women, which was not significantly different. Maximal principal strain and radial strain showed the greatest elevation in global peak systolic amplitude and systolic strain rate, with a relative increase that exceeded 10% versus healthy volunteers. |
| van der Velde N et al. [11] | 2021 | 80 patients and 40 controls | 47 ± 11 | 46 (patients) 47 (control) | lymphoma | mediastinal radiotherapy with or without anthracyclines | GLS (−19.5% ± 2.5% vs −20.6% ± 2.0%; P = 0.013), GCS (−17.9% ± 2.5% vs −20.4% ± 2.2%; P < 0.001), and GRS (69% ± 15% vs 76% ± 15%; P = 0.018) of the LV were reduced in lymphoma survivors. |
| Calvillo-Argüelles O et al. [12] | 2022 | 136 patients | 51.1 ± 9.2 | 100 | breast cancer | anthracycline and trastuzumab | An absolute change in GLS (standardized odds ratio [sOR]: 1.97 [95% CI: 1.07-3.66]; P = 0.031) was associated with concurrent cancer treatment-related cardiac dysfunction. An absolute change in GLS (sOR: 1.79 [95% CI: 1.22-2.62]; P = 0.003) was associated with subsequent cancer treatment-related cardiac dysfunction. |
| Mega S et al. [13] | 2022 | 34 patients | 69.5 (43–87) | 23.5 | non-small cell lung cancer | chemoradiotherapy | GLS and EF progressively decreased from baseline to M1 and M3. There was a strong correlation between GLS and EF reduction (at M1: p = 0.034; at M3: p = 0.018). |
| Cheng S et al. [14] | 2023 | 24 patients | 47.2 ± 11.1 | 100 | breast cancer | anti-HER2 therapy and nonanthracycline-based chemotherapy | GLS decreased significantly at follow-up in anti-HER2 group and trastuzumab and pertuzumab group. The decrease in GLS in the trastuzumab group was not significant (p = 0.169). |
| Kar J et al. [15] | 2023 | 32 patients | 59.4 (9.7) | 100 | breast cancer | anthracycline, cyclophosphamide, and taxol | GLS worsened from baseline to the 3- and 6-month follow-ups (-19.1 ± 2.1%, -16.0 ± 3.1%, -16.1 ± 3.0%; P < 0.001). Univariable Cox regression showed the 3-month GLS significantly associated as an agonist for cancer therapy-related cardiac dysfunction occurrence. Bivariable regression showed the 3-month GLS as a cancer therapy-related cardiac dysfunction prognostic factor independent of other covariates. |
| Thavendiranathan P et al. [16] | 2023 | 136 patients | 51.1 (9.2) | 100 | stage I to III ERBB2-positive breast cancer | anthracyclines and trastuzumab with/without adjuvant radiotherapy and surgery | Nadir LVEF, GLS, and GCS levels were seen between 3 and 6 months after trastuzumab initiation coinciding with the largest LV end-systolic and end-diastolic volume index and LV mass index |
| Zheng Y et al. [17] | 2023 | 58 patients | 52.82 ± 2.61 | 100 | breast cancer | anthracycline | GLS and strain rate decreased signiﬁcantly at the second, fourth, and sixth cycle of chemotherapy compared to baseline, but no statistically signiﬁcant differences were observed in GCS, GRS, GCS-s and GRS-s(P = 0.26, 0.13, 0.13, and 0.10, respectively). ΔGLS and MΔGLS-s were significantly associated with the chemotherapy cycle (Pearson’s correlation coefﬁcients for GLS = 0.75; Spearman’s correlation coefﬁcients for GLS-s = 0.75) |
| Beitzen-Heineke A et al. [18] | 2024 | 44 patients and 21 controls | 44(37-52) (patients) 44(30-52) (controls) | 0 | germ cell cancer | platinum | The strain analysis revealed significantly reduced deformation compared to controls (LV global longitudinal strain [GLS] -13 ± 2% vs. -15 ± 1%, p < 0.001; RV GLS -15 ± 4% vs. -19 ± 4%, p = 0.005). |
| Kersten J et al. [19] | 2024 | 34 patients and 10 controls | 50.2 ± 10.3 | 100 | breast cancer | anthracycline | Left ventricular GRS (p = 0.008), GCS (p = 0.010), and GLS (p = 0.036) were reduced at follow-up. Twelve months after cancer diagnosis, the breast cancer patients exhibited significant impairments in left ventricular GRS (p = 0.001), GCS (p = 0.001), and GLS (p = 0.002) compared to the healthy controls. |

2D, two dimensional; 2DE, two dimensional echocardiography; 3D, three dimensinal; CMR, cardiac magnetic resonance; CTRCD, cancer therapy related cardiac dysfunction; FT, feature-tracking; GCS, global circumferential strain; GLS, global longitudinal strain; GRS, global radical strain; LV, left ventricular; LVEF, left ventricular ejection fraction.

**Reference：**

1. Drafts BC, et al., *Low to moderate dose anthracycline-based chemotherapy is associated with early noninvasive imaging evidence of subclinical cardiovascular disease.* JACC Cardiovasc Imaging, 2013. **6**(8): p. 877-85.

2. Nakano S, et al., *Cardiac magnetic resonance imaging-based myocardial strain study for evaluation of cardiotoxicity in breast cancer patients treated with trastuzumab: A pilot study to evaluate the feasibility of the method.* Cardiol J, 2016. **23**(3): p. 270-80.

3. Jolly MP, et al., *Automated assessments of circumferential strain from cine CMR correlate with LVEF declines in cancer patients early after receipt of cardio-toxic chemotherapy.* J Cardiovasc Magn Reson, 2017. **19**(1): p. 59.

4. Ong G, et al., *Myocardial strain imaging by cardiac magnetic resonance for detection of subclinical myocardial dysfunction in breast cancer patients receiving trastuzumab and chemotherapy.* Int J Cardiol, 2018. **261**: p. 228-233.

5. Gong IY, et al., *Early diastolic strain rate measurements by cardiac MRI in breast cancer patients treated with trastuzumab: a longitudinal study.* Int J Cardiovasc Imaging, 2019. **35**(4): p. 653-662.

6. Lambert J, et al., *Variability in echocardiography and MRI for detection of cancer therapy cardiotoxicity.* Heart, 2020. **106**(11): p. 817-823.

7. Houbois CP, et al., *Serial Cardiovascular Magnetic Resonance Strain Measurements to Identify Cardiotoxicity in Breast Cancer: Comparison With Echocardiography.* JACC Cardiovasc Imaging, 2021. **14**(5): p. 962-974.

8. Barbosa MF, et al., *Characterization of subclinical diastolic dysfunction by cardiac magnetic resonance feature-tracking in adult survivors of non-Hodgkin lymphoma treated with anthracyclines.* BMC Cardiovasc Disord, 2021. **21**(1): p. 170.

9. Bouwer NI, et al., *2D-echocardiography vs cardiac MRI strain: a prospective cohort study in patients with HER2-positive breast cancer undergoing trastuzumab.* Cardiovasc Ultrasound, 2021. **19**(1): p. 35.

10. Labib D, et al., *Effect of Active Cancer on the Cardiac Phenotype: A Cardiac Magnetic Resonance Imaging-Based Study of Myocardial Tissue Health and Deformation in Patients With Chemotherapy-Naïve Cancer.* J Am Heart Assoc, 2021. **10**(9): p. e019811.

11. van der Velde N, et al., *Detection of Subclinical Cardiovascular Disease by Cardiovascular Magnetic Resonance in Lymphoma Survivors.* JACC CardioOncol, 2021. **3**(5): p. 695-706.

12. Calvillo-Argüelles O, et al., *Diagnostic and Prognostic Value of Myocardial Work Indices for Identification of Cancer Therapy-Related Cardiotoxicity.* JACC Cardiovasc Imaging, 2022. **15**(8): p. 1361-1376.

13. Mega S, et al., *Early GLS changes detection after chemoradiation in locally advanced non-small cell lung cancer (NSCLC).* Radiol Med, 2022. **127**(12): p. 1355-1363.

14. Cheng S, et al., *Longitudinal assessment of cardiac parameters through MRI in breast cancer patients treated with anti-HER2 therapy.* Eur Radiol Exp, 2023. **7**(1): p. 22.

15. Kar J, et al., *Can global longitudinal strain (GLS) with magnetic resonance prognosticate early cancer therapy-related cardiac dysfunction (CTRCD) in breast cancer patients, a prospective study?* Magn Reson Imaging, 2023. **97**: p. 68-81.

16. Thavendiranathan P, et al., *Comprehensive Cardiovascular Magnetic Resonance Tissue Characterization and Cardiotoxicity in Women With Breast Cancer.* JAMA Cardiol, 2023. **8**(6): p. 524-534.

17. Zheng Y, et al., *Serial Cardiac MRI for Quantification of the Dynamics of Anthracycline-Induced Subclinical Myocardial Injury.* J Magn Reson Imaging, 2023. **58**(5): p. 1533-1541.

18. Beitzen-Heineke A, et al., *Long-term cardiotoxicity in germ cell cancer survivors after platinum-based chemotherapy: cardiac MR shows impaired systolic function and tissue alterations.* Eur Radiol, 2024. **34**(6): p. 4102-4112.

19. Kersten J, et al., *CMR reveals myocardial damage from cardiotoxic oncologic therapies in breast cancer patients.* Int J Cardiovasc Imaging, 2024. **40**(2): p. 225-235.
